# Cerebrospinal Fluid Biomarkers of Myeloid and Glial Cell Activation Are Correlated With Multiple Sclerosis Lesional Inflammatory Activity

**DOI:** 10.3389/fnins.2021.649876

**Published:** 2021-03-30

**Authors:** Ruturaj Masvekar, Jonathan Phillips, Mika Komori, Tianxia Wu, Bibiana Bielekova

**Affiliations:** ^1^National Institute of Allergy and Infectious Diseases, National Institutes of Health, Bethesda, MD, United States; ^2^National Institute of Neurological Disorders and Stroke, NIH, Bethesda, MD, United States

**Keywords:** multiple sclerosis, cerebrospinal fluid biomarkers, contrast-enhancing lesions, lesional inflammatory activity, axonal damage

## Abstract

Multiple sclerosis (MS)-related inflammation can be divided into lesional activity, mediated by immune cells migrating from the periphery to the central nervous system (CNS) and non-lesional activity, mediated by inflammation compartmentalized to CNS tissue. Lesional inflammatory activity, reflected by contrast-enhancing lesions (CELs) on the magnetic resonance imaging (MRI), is effectively inhibited by current disease modifying therapies (DMTs). While, the effect of DMTs on non-lesional inflammatory activity is currently unknown. Reliable and simultaneous measurements of both lesional and non-lesional MS activity is necessary to understand their contribution to CNS tissue destruction in individual patients. We previously demonstrated that CNS compartmentalized inflammation can be measured by combined quantification of cerebrospinal fluid (CSF) immune cells and cell-specific soluble markers. The goal of this study is to develop and validate a CSF-biomarker-based molecular surrogate of MS lesional activity. The training cohort was dichotomized into active (CELs > 1 or clinical relapse) and inactive lesional activity (no CELs or relapse) groups. Matched CSF and serum samples were analyzed for 20 inflammatory and axonal damage biomarkers in a blinded fashion. Only the findings from the training cohort with less than 0.1% probability of false positive (i.e., *p* < 0.001) were validated in an independent validation cohort. MS patients with lesional activity have elevated IL-12p40, CHI3L1, TNFα, TNFβ, and IL-10, with the first two having the strongest effects and validated statistically-significant association with lesional activity in an independent validation cohort. Marker of axonal damage, neurofilament light (NfL), measured in CSF (cNfL) was also significantly elevated in MS patients with active lesions. NfL measured in serum (sNfL) did not differentiate the two MS subgroups with pre-determined significance, (*p* = 0.0690) even though cCSF and sNfL correlated (Rho = 0.66, *p* < 0.0001). Finally, the additive model of IL12p40 and CHI3L1 outperforms any biomarker discretely. IL12p40 and CHI3L1, released predominantly by immune cells of myeloid lineage are reproducibly the best CSF biomarkers of MS lesional activity. The residuals from the IL12p40/CHI3L1-cNfL correlations may identify MS patients with more destructive inflammation or contributing neurodegeneration.

## Introduction

The multiple sclerosis (MS) lesional inflammatory activity is associated with blood brain barrier (BBB) breakdown and transmigration of immune cells from periphery to central nervous system (CNS) (Lassmann, 2018). The lesional inflammatory activity can be measured by contrast-enhancing lesions (CELs) on brain magnetic resonance imaging (MRI). The measurement of lesional activity via CELs is non-invasive and convenient, and thus utilized as an outcome in most phase II trials of immunomodulatory treatments for MS (Sormani and Bruzzi, 2013; Filippi et al., 2019). However, the broad use of CELs in the MS field led to a false generalization that CELs represent all MS-related inflammatory activities in the CNS. CELs only reflect the perivascular inflammation associated with the opening of the BBB and influx of immune cells from blood to form MS lesion. We call this “lesional” inflammation. But there are other inflammatory processes in MS that are not captured by CELs, such as formation of cortical lesions not typically associated with BBB opening and more diffuse inflammation compartmentalized to CNS tissue, often seen in progressive MS. We call this “non-lesional” inflammation (Frischer et al., 2009; Androdias et al., 2010; Komori et al., 2015; Milstein et al., 2019).

The current disease modifying therapies (DMTs) effectively inhibit formation of focal lesions (Buron et al., 2020), but their effect on non-lesional inflammatory activities is unknown. Understanding contributions of lesional versus non-lesional MS inflammatory activity to CNS tissue destruction requires reliable and simultaneous measurements of both processes in the same patients. While measuring lesional activity via CELs using MRI is non-invasive and convenient, there are limitations to this measurement: (a) Most often, only brain CELs are measured, but inflammation and BBB breakdown occurs in the spinal cord too (Moccia et al., 2019); (b) Most common CEL measurement is only semi-quantitative, representing the number of unique CELs, but not their volume; and (c) CEL detection is also dependent on the dose of the contrast administered and the length of time that elapsed between contrast administration and the image acquisition. CSF biomarkers can’t compete with the convenience of repeated MRIs for monitoring MS lesional activity; however, they can be used in research settings or in diagnostic lumbar puncture (LP) to measure contributions of lesional versus non-lesional MS inflammation to axonal damage in individual patients.

We have previously devised methodology that allows measurement of CNS compartmentalized inflammation in living human subjects without a need for CNS tissue biopsy, using combination of CSF cellular, and molecular biomarkers (Komori et al., 2015). This method relies on soluble biomarkers that are exclusively, or predominantly released from a specific immune cell type, such as sCD27 (mostly released by T cells, CD8 > CD4), sCD21 (released by B cells, naive > memory) and sCD14 (released by monocytes and possibly microglia). The ratio of these biomarkers to the cells of their origin (measured in CSF via flow cytometry) in healthy volunteers who have no compartmentalized inflammation, represents release of these cell-specific markers by CSF immune cells. If there is excess of cell-specific biomarkers in comparison to the CSF cells of their origin, then there must be other sources of immune cells, that are not in the CSF, but can release their biomarkers into CSF. Brain pathology demonstrated that these additional cells are in CNS tissue, representing compartmentalized inflammation, or non-lesional MS inflammatory activity.

Thus, the next step is to analyze multiple candidate CSF biomarkers of MS lesional activity to determine which of them is most accurate and whether they can be combined into a model that outperforms the single best CSF biomarker. We then thought to assess the strength of correlation between the winning CSF surrogate of MS lesional activity and neurofilament light chain (NfL), a validated marker of axonal damage (Norgren et al., 2004; Teunissen and Khalil, 2012; Gaetani et al., 2019; Alirezaei et al., 2020). Such CSF-biomarker-based differentiation of lesional and non-lesional inflammation from the identical CSF samples will allow for assessing the contribution of these two processes to CNS tissue destruction in MS.

Neurofilaments are important cytoskeletal proteins of axons; in contrast to other cytoskeletal proteins neurofilaments are specific to neurons. Damage of axons during various neurodegenerative diseases leads to releases of neurofilaments into interstitial fluid and finally into CSF and blood. As NfL has the lowest molecular weight of the three neurofilament subunits (light, medium, and heavy), it diffuses more easily from parenchyma to CSF after axonal damage or neuronal death (Fuchs and Cleveland, 1998; Scherling et al., 2014; Alirezaei et al., 2020). Thus, NfL is a reliable biomarker of axonal damage.

NfL concentration in CSF is approximately 100-fold higher than in the blood. But with advent of highly sensitive assays (e.g., single molecular array, SIMOA) (Rissin et al., 2010; Kan et al., 2012). NfL concentration in blood also can be measured reliably. As collection of blood is easier and less invasive compared to CSF, blood NfL is being commonly examined as a biomarker for axonal damage in neurological diseases. Thus, to measure the relationship between lesional MS activity and axonal damage, we measured NfL concentrations both in CSF (cNfL) and blood/serum (sNfL).

## Materials and Methods

### Research Subjects

Subjects were prospectively recruited to the NIH Institutional Review Board (IRB)-approved protocol “Comprehensive Multimodal Analysis of Neuroimmunological Diseases of the CNS” (ClinicalTrials.gov Identifier: NCT00794352), between 07/2003 to 05/2019.

The inclusion criteria for healthy donors (HD): (1) at least 18 years old at time of enrollment, (2) vital signs within normal range at time of screening visit, and (3) able to give informed consent and undergo required research procedures. The exclusion criteria for HD: (1) previous or current history of alcohol or substance abuse, (2) inflammatory or non-inflammatory neurological diseases, (3) medical contraindications with required research procedures, and (4) pregnancy or current breastfeeding.

The inclusion criteria for patients: (1) at least 12 years old at time of enrollment, (2) presentation with a clinical syndrome consistent with immune-mediated CNS disorder and/or neuroimaging evidence of inflammatory and/or demyelinating CNS disease, (3) adults able to give informed consent on their own or via legally authorized representative and minors willing to assent and able to give informed consent via parents or legal guardian, and (4) able to undergo required research procedures. The exclusion criteria for patients: (1) significant medical conditions that would make participation in diagnostic or research part of evaluation impossible or risky, (2) unable or unwilling to give informed consent, and (3) medical contraindications with required research procedures.

All subjects underwent neurological examination to derive the measures of neurological disability Expanded Disability Status Scale (EDSS) (Kurtzke, 1983). Composite MRI scale of CNS tissue destruction (COMRIS-CTD) was calculated from 3T brain MRI images as described (Kosa et al., 2015). MS diagnostic subgroups (relapsing remitting MS [RR-MS], secondary progressive MS [SP-MS] and primary progressive MS [PP-MS]) were classified using McDonald’s criteria, 2010 and 2017 revisions (Polman et al., 2011; Thompson et al., 2018). MS patients were divided into two subgroups based on presence or absence of lesional inflammatory activity determined by recognition of CELs on MRI using clinical-grade structural MRI images of the brain collected under a published protocol (Kosa et al., 2015). The CELs were recognized on co-registered images as hyperintense on T2WI/FLAIR, hypo-or iso-intense on T1WI and hyperintense on post-contrast T1WI (3D-GRE or 3D-FSPGE-BRAVO).

The findings from the training cohort (70 MS patients) that had less than 0.1% probability of false positive (i.e., unadjusted *p* < 0.001) were then validated in an independent validation cohort (130 MS patients); Table 1 represents demographic details of subjects from both cohorts.

**TABLE 1:** Demographic details of training and validation cohorts. Across MS lesional activity subgroups (inactive versus active) categorical variables (gender and MS disease type distributions) were compared using Chi-square test (^∗^) and continuous variables (age, disease duration, and EDSS) were compared using *t*-test (^#^).

				**MS Lesional Activity**		
			**HD**	**Inactive**	**Active**	***p***
Training cohort	N		5	35	35	
	Gender	(Female/male)	4/1	16/19	22/13	0.1500*
	MS type	(RR-MS/SP-MS/PP-MS)		7/15/13	28/7/0	<0.0001*
	Age	Mean (SD)	47.5 (14.4)	52.8 (11.3)	37.6 (10.8)	<0.0001^#^
	Disease duration	Mean (SD)		13.7 (10.8)	5.1 (7.4)	0.0002^#^
	EDSS	Mean (SD)		5.1 (1.9)	2.7 (2.1)	<0.0001^#^
Validation cohort	N		13	96	34	
	Gender	(Female/male)	6/7	50/46	19/15	0.7029*
	MS type	(RR-MS/SP-MS/PP-MS)		42/26/28	24/5/5	0.0267*
	Age	Mean (SD)	37.5 (10.8)	49.4 (10.4)	41.0 (13.2)	0.0003^#^
	Disease duration	Mean (SD)		12.5 (9.5)	6.3 (8.6)	0.0009^#^
	EDSS	Mean (SD)		3.7 (2.2)	2.7 (2.1)	0.0229^#^

### CSF and Blood Sample Collection and Processing

CSF and blood samples were collected according to standard operating procedures as described (Masvekar et al., 2019). CSF samples were collected by LP and stored on ice until further processing. Research CSF aliquots were centrifuged at 1,200 rpm for 10 min at 4°C to pellet out cells. Then cell-free CSF supernatants were aliquoted (0.5 ml/vial) and stored at −80°C until further use. Blood samples were collected in serum separation tubes (SST^TM^, BD Vacutainer^TM^, Thermo Fisher Scientific, Waltham, MA, United States) and centrifuged at 3000 rpm for 10 min at 4°C. Then serum supernatants were aliquoted (0.5 ml/vial) and stored at −80°C until further use. Frozen CSF and serum samples were thawed on ice and used for biomarker analyses; repeated freezing and thawing of biological samples was avoided.

### Biomarker Analyses

CSF and serum samples were analyzed using single molecule arrays (Simoa^TM^) assay kits (Quanterix^TM^, Billerica, MA, United States) or spectrophotometric ELISA kits (UmanDiagnostics, Umea, Sweden) or homebrew electrochemiluminescence (ECL) ELISAs using the Meso Scale Discovery detection system (MSD; Rockville, MD, United States) (Table 2).

**TABLE 2:** List of kits and antibodies used for biomarker analyses using either Simoa (Quanterix) or spectrophotometric or homebrew MSD-ECL ELISAs; working concentrations or dilutions of coating and detection antibodies for homebrew assays and lower limit of detection (LLoD) of all assays is provided here. IL-12p40 in training (^∗^) and validation (^#^) cohort was analyzed using Simoa and MSD-ECL ELISA, respectively.

**Biomarkers**	**ELISA Type**	**Kit or antibodies source**	**Working concentrations/dilutions of coating and detection antibodies**	**LLoD**
TNFα	Simoa^TM^	Quanterix (101580)		0.016 pg/ml
IL-1β	Simoa^TM^	Quanterix (101605)		0.016 pg/ml
TNFβ	Simoa^TM^	Quanterix (102091)		0.052 pg/ml
LIF	Simoa^TM^	Quanterix (102394)		0.015 pg/ml
TRAIL	Simoa^TM^	Quanterix (100906)		0.0083 pg/ml
GM-CSF	Simoa^TM^	Quanterix (102329)		0.0019 pg/ml
IL-10	Simoa^TM^	Quanterix (101643)		0.0038 pg/ml
TGFβ	Simoa^TM^	Quanterix (101984)		0.137 pg/ml
IL-17F	Simoa^TM^	Quanterix (102082)		1.08 pg/ml
IL-12p40*	Simoa^TM^	Quanterix (101871)		0.02 pg/ml
IL-12p40^#^	MSD-ECL	Mesoscale Diagnostics (K15050D)		4.6 pg/ml
sNfL	Simoa^TM^	Quanterix (103186)		0.038 pg/ml
cNfL	Spectrophotometric	UmanDiagnostics (10-7002)		100 pg/ml
SERPINA3	Homebrew MSD-ECL	R&D Systems (MAB1295 and BAF1295)	1 μg/ml and 250 ng/ml	125 ng/ml
CXCL13	Homebrew MSD-ECL	R&D Systems (DY801)	1 μg/ml and 100 ng/ml	17.4 pg/ml
CD27	Homebrew MSD-ECL	Sanquin (M1960)	1:100 and 1:100	0.4 U/ml
CD14	Homebrew MSD-ECL	R&D Systems (DY383)	2 μg/ml and 100 ng/ml	0.49 ng/ml
BAFF	Homebrew MSD-ECL	R&D Systems (DY124-05)	500 ng/ml and 50 ng/ml	7 pg/ml
CD21	Homebrew MSD-ECL	R&D Systems (DY4909-05)	250 ng/ml and 100 ng/ml	5.8 pg/ml
BCMA	Homebrew MSD-ECL	R&D Systems (DY193)	400 ng/ml and 400 ng/ml	7 pg/ml
CHI3L1	Homebrew MSD-ECL	R&D Systems (DY2599)	4 μg/ml and 100 ng/ml	41 pg/ml

Assay kits were used as per manufacturer’s instructions. For homebrew assay development, we used a published protocol (Komori et al., 2015). Briefly, MSD bare plates (MULTI-ARRAY 96-Well Plate; Catalog# L15XA-3) were coated with working concentrations of capture antibody overnight at 4°C. Coating solution was aspirated, plates were washed with phosphate buffer saline (PBS)-Tween 20 (PBS-T) and then incubated with 1% bovine serum albumin (BSA) in PBS for 2 h at room temperature. After washing, working dilutions of standards and samples were added to the plate and incubated for 2 h at room temperature. Plates were washed and incubated with working concentrations of biotinylated-detection antibody for 2 h at room temperature. After washing, plates were incubated with 0.25 μg/ml of SULFO-TAG^TM^ streptavidin (MSD, Catalog# R32AD-1) in 1% BSA/PBS for 1 h at room temperature. Finally, plates were washed and added 2X Read Buffer (MSD, Catalog# R92TC-1), and then ECL was analyzed using QuickPlex SQ 120 (MSD).

All samples were analyzed in blinded fashion and in duplicates, results were accepted only when coefficient of variance (CV) across the sample duplicates was <20%. Samples were analyzed on multiple plates, location of samples on each plate were randomized. On each plate a control sample was analyzed in duplicate; The CV for the control sample across the plates is <20%, confirming the assay precision and reproducibly.

### Adjustment for Effect of Healthy Aging

Some biomarkers analyzed in this study are known to change with age in healthy subjects. Specifically, cNfL (Vågberg et al., 2015), sNfL (Disanto et al., 2017) and CHI3L1 (Bonneh-Barkay et al., 2010) have shown to be correlated with age within healthy subjects. As in this study, age of MS subjects across lesional activity subgroups is significantly different (Table 1; inactive versus active), it is essential to adjust for the effect of healthy aging. To derive adjustment equations, we pooled all cNfL, sNfL, and CHI3L1 HD subjects’ data available in our research database (Table 3 and Supplementary Data File 1). As described previously (Barbour et al., 2020), linear regression models for the logarithmic value of biomarker concentrations with age as an independent variable were used to predict the healthy, age-related levels of these biomarkers for all MS patients according to their age at time of sample collection; Then to exclude the effect of healthy aging, these predicted biomarker levels due to healthy aging were subtracted from true, measured biomarker levels.

**TABLE 3:** Demographic details of healthy cohort, used to derive linear regression models for the biomarker concentrations (cNfL, sNfL, and CHI3L1) with age as an independent variable.

		**cNfL**	**sNfL**	**CHI3L1**
*N*		78	26	68
Gender	(Female/male)	39/39	14/12	37/31
Age	Mean (SD)	40.8 (12.6)	38.9 (15.7)	41.3 (12.8)
	Range	19.4–71.3	19.4–71.3	19.4–71.3

### Data Transformation and Statistical Analyses

Data were organized using Microsoft Excel (Microsoft, Redmond, WA, United States); all biomarker concentration data for both cohorts (training and validation) is provided in Supplementary Data File 1. All biomarkers were compared across MS lesional activity subgroups (inactive versus active) using unpaired *t*-test.

Correlations between biomarkers and number of CELs were evaluated using Spearman coefficient, and multiple linear regression analyses. For normality assumption of regression analysis, natural logarithm transformation was applied to some biomarkers (cNfL, sNfL, IL-12p40, CHI3L1, and CXCL13). As concentrations of cNfL, sNfL, and CHI3L1 were adjusted for effect of healthy aging some patients may have negative adjusted values; So, before applying natural logarithm transformation to age-adjusted concentrations these values were mathematically transformed to positive by adding “minimum value +1.”

Principal component analysis was performed using the biomarkers showing significant correlation with number of CELs. The first component (a linear combination of the biomarkers) was used to predict the lesional activity.

GraphPad Prism 7 (GraphPad Software Inc., La Jolla, CA, United States) and SAS 9.4 (SAS Institute Inc.) were used for the data analyses.

## Results

### Effect of Healthy Aging on Biomarker Concentrations

**FIGURE 1:**
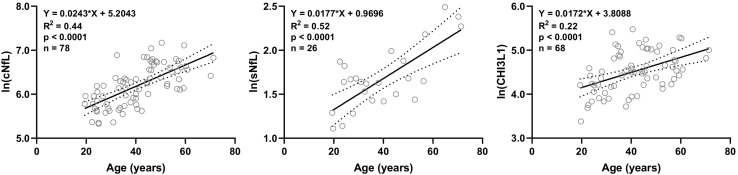
In HD subjects’ research database, linear regression of CSF and serum NfL (cNfL and sNfL) and CHI3L1 with age as an independent variable was analyzed; All three biomarkers were significantly (*R*^2^ = 0.44, 0.52 and 0.22, respectively, and *p* < 0.0001) correlated with age.

Out of 20 biomarkers analyzed, only 3 biomarkers showed statistically significant correlations with age in HD subjects’ data available in our research database: cNfL (*n* = 78, R^2^ = 0.44, 95% CI = 0.018–0.031, and *p* < 0.0001), sNfL (*n* = 26, R^2^ = 0.52, 95% CI = 0.010–0.025, and *p* < 0.0001) and CHI3L1 (*n* = 68, R^2^ = 0.22, 95% CI = 0.009–0.025, and *p* < 0.0001) (Figure 1).

Linear regression models of cNfL (ln[cNfL] = 0.0243^∗^Age + 5.2043), sNfL (ln[sNfL] = 0.0177^∗^Age + 0.9696) and CHI3L1 (ln[CHI3L1] = 0.0172^∗^Age + 3.8088) with age as an independent variable were used to predict the healthy, age-related levels of these biomarkers for all MS patients according to their age at time of sample collection.

### Analysis of Proinflammatory Biomarkers Across MS Lesional Activity Subgroups

**FIGURE 2:**
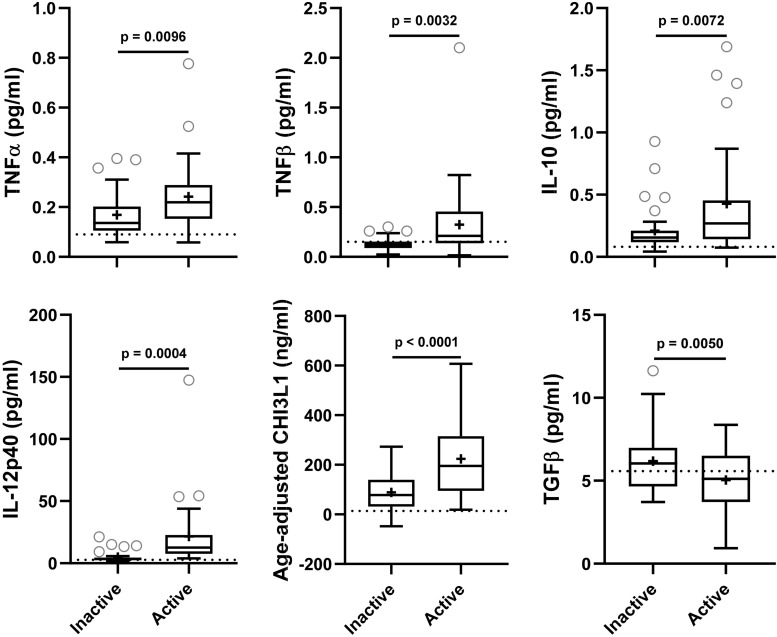
In the training cohort, biomarker concentrations were compared across lesional activity subgroups (inactive versus active, *n* = 35 each subgroup). Dotted line represents median of HDs and “+” sign represents mean of respective subgroup. Data only for biomarkers which are statistically significantly different (*p* < 0.01; unpaired *t*-test) between two subgroups is depicted here.

Biomarkers were analyzed in subjects’ CSF samples in blinded manner. After unblinding categorization of MS patients into active versus inactive groups, TNFα, TNFβ, IL-10, IL-12p40, and CHI3L1 (unadjusted *p* = 0.0096, 0.0032, 0.0072, 0.0004, and <0.0001, respectively) were significantly elevated in CSF of MS patients with active lesional activity (CELs > 1 or in clinical relapse) compared to patients with inactive lesional activity (CELs = 0 or not in clinical relapse). While, TGFβ (unadjusted *p* = 0.0050) was significantly downregulated in active MS subjects (Table 4 and Figure 2).

**TABLE 4:** In the training cohort, biomarker concentrations across HD (*n* = 5) and MS lesional activity subgroups (Inactive and Active, *n* = 35 each subgroup) are represented as median (range).

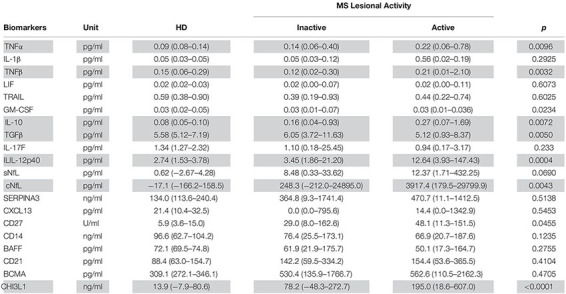

*Biomarker concentrations were compared between inactive and active subgroups using unpaired *t*-test; Only biomarkers that are statistically significantly different (*p* < 0.01) between these subgroups are highlighted.*

In the training cohort, MS disease type (RR-MS and P-MS, representing both primary[PP-MS]- and secondary [SP-MS]-progressive MS) distribution across lesional activity subgroups (active vs inactive) is significantly different (Table 1; Chi-square test, *p* < 0.0001). Thus, significantly (*p* < 0.01) different biomarkers within lesional activity subgroups were compared across MS disease type (RR-MS vs P-MS) within respective lesional activity subgroups (active and inactive) using unpaired t-test (Supplementary Figure 1). None of these biomarkers were statistically significantly (*p* < 0.05) different between MS disease types within respective lesional activity subgroup. These findings suggest that observed variability in biomarkers across lesional activity subgroups (active vs inactive) is not driven by variability in MS disease type distribution.

### Analysis of Axonal Damage Biomarkers Across MS Lesional Activity Subgroups

**FIGURE 3:**
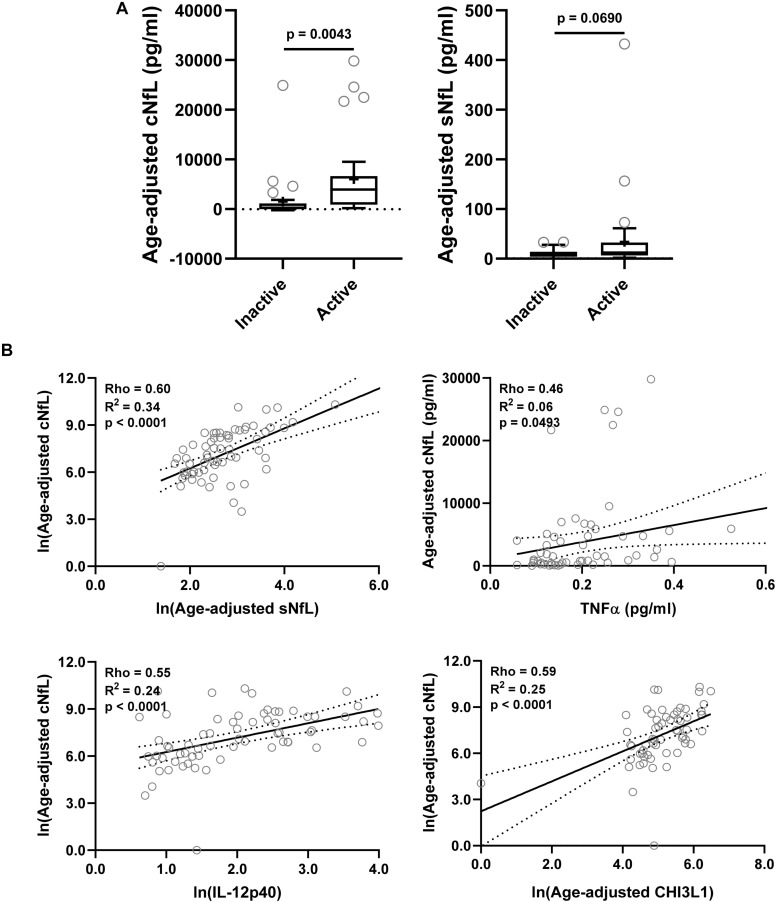
In the training cohort, **(A)** CSF and serum NfL (cNfL and sNfL) concentrations were compared across MS lesional activity subgroups (*n* = 35 each subgroup). The median concentration of HDs is represented with a dotted line, and the mean for respective subgroups is represented with “+” sign. cNfL was significantly elevated in MS patients with active lesional activity (*p* < 0.0043; unpaired *t*-test). However, though sNfL was elevated in active MS patients it did not reach predefined statistical significance (*p* = 0.0690). **(B)** Within MS patients (*n* = 70) correlations between cNfL and all other biomarkers were analyzed; Only for statistically significantly (*p* < 0.01; Spearman correlation analysis) correlated biomarkers linear regression with cNfL as a dependent variable was analyzed. Solid line represents “line of best fit,” and dotted line represents 95% confidence intervals.

NfL a marker of axonal damage (Norgren et al., 2004; Teunissen and Khalil, 2012; Gaetani et al., 2019; Alirezaei et al., 2020), was analyzed in patients’ CSF and serum samples, and then compared across MS lesional activity subgroups. In MS patients with active lesional activity CSF NfL (cNfL) level was significantly elevated compared to patients without lesional inflammatory activity (unadjusted *p* = 0.0043; Table 4 and Figure 3A). Though there is a strong correlation between NfL levels in serum and CSF (Spearman Rho = 0.60, *R*^2^ = 0.34 and *p* < 0.0001; Figure 3B and Supplementary Table 1), sNfL is not statistically significantly different between active and inactive MS subgroups (unadjusted *p* = 0.0690; Table 4, and Figure 3A).

Correlations between cNfL and other biomarkers were analyzed using Spearman analysis; for statistically significantly (*p* < 0.01) correlated biomarkers linear regression with cNfL as a dependent variable was analyzed: sNfL (Rho = 0.60, *R*^2^ = 0.34, and *p* < 0.0001), TNFα (Rho = 0.46, *R*^2^ = 0.06, and *p* = 0.0493), IL-12p40 (Rho = 0.55, *R*^2^ = 0.24, and *p* < 0.0001) and CHI3L1 (Rho = 0.59, *R*^2^ = 0.25, and *p* < 0.0001) (Figure 3B and Supplementary Table 1).

### Correlations Between Biomarkers and Number of CELs

**FIGURE 4:**
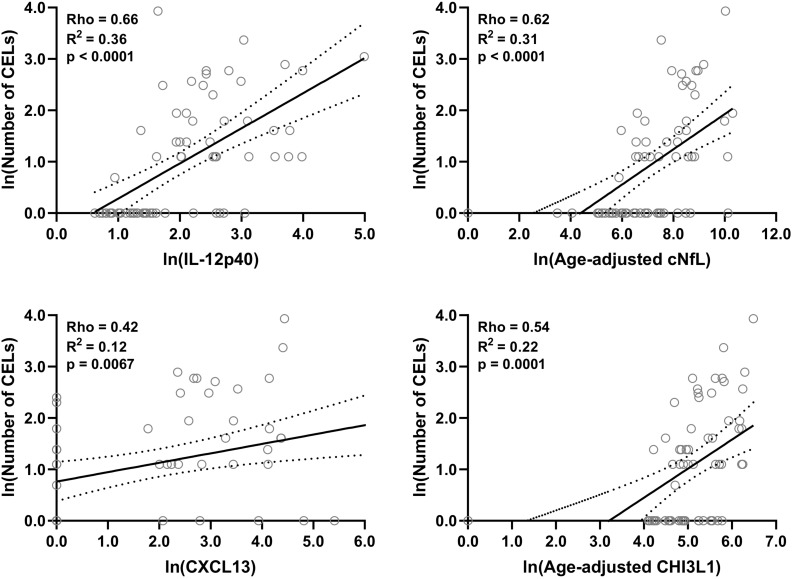
In the training cohort, within MS patients (*n* = 70) correlations between number of CELs and all biomarkers were analyzed; Only for statistically significantly (*p* < 0.01; Spearman correlation analysis) correlated biomarkers linear regression with number of CELs as a dependent variable was analyzed. Solid line represents “line of best fit,” and dotted line represents 95% confidence intervals.

Within both (inactive and active) type of MS patients, correlations between number of CELs and all other biomarkers were analyzed using Spearman analysis; And then only for statistically significantly (*p* < 0.01) correlated biomarkers linear regression with number of CELs as a dependent variable was analyzed: IL-12p40 (Rho = 0.66, *R*^2^ = 0.36, and *p* < 0.0001), cNfL (Rho = 0.62, *R*^2^ = 0.31, and *p* < 0.0001), CXCL13 (Rho = 0.42, *R*^2^ = 0.12, and *p* = 0.0067) and CHI3L1 (Rho = 0.54, *R*^2^ = 0.22, and *p* = 0.0001) (Figure 4 and Supplementary Table 2).

### Combined Model of Biomarkers

We tried to develop a stronger molecular model to predict lesional activity via combining several biomarkers together using the first principal component. But none of the principal component scores outperformed individual IL-12p40 and CHI3L1 in differentiating between active versus inactive subgroups.

In order to get a combined model of biomarkers, for two strongly correlated lesional inflammatory biomarkers with cNfL, IL-12p40, and CHI3L1, the multiple linear regression analysis with cNfL as a dependent variable was performed. The multiple linear regression analysis yields a model (ln[cNfL] = 2.403 + 0.6643^∗^ln[IL-12p40] + 0.6747^∗^ln[CHI3L1]) which outperforms (*R*^2^ = 0.35 and *p* < 0.0001) any biomarker individually.

Similarly, multiple linear regression analysis of IL-12p40, CHI3L1, and cNfL with the number of CELs as a dependent variable was performed (ln[Number of CELs] = −2.161 + 0.3865^∗^ln[IL-12p40] + 0.1972^∗^ln[CHI3L1] + 0.1938^∗^ln[cNfL]). This model performs (*R*^2^ = 0.45 and *p* < 0.0001) better than any single biomarker.

### Analysis of Correlations Between Biomarkers

**FIGURE 5:**
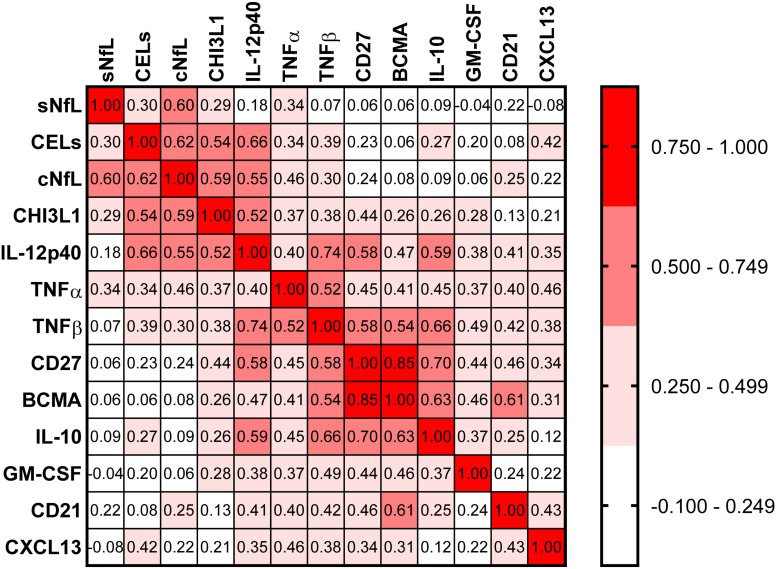
In the training cohort, correlations between all biomarkers were analyzed using Spearman analysis. In correlogram, the color intensity of each cell denotes the correlation coefficient (Spearman Rho) for correlation between respective biomarkers. All biomarkers were analyzed, but only biomarkers which have at least one statistically significant (*p* < 0.01) correlation are depicted here.

Correlations between all biomarkers were analyzed using Spearman analysis (Figure 5). In a correlation matrix a strong cluster of four proinflammatory biomarkers (CHI3L1, IL-12p40, TNFα, and TNFβ) can be identified; these four proinflammatory biomarkers were significantly elevated in active MS patients, and they were strongly correlated with axonal damage (cNfL) and number of CELs.

### Validation Cohort

**FIGURE 6:**
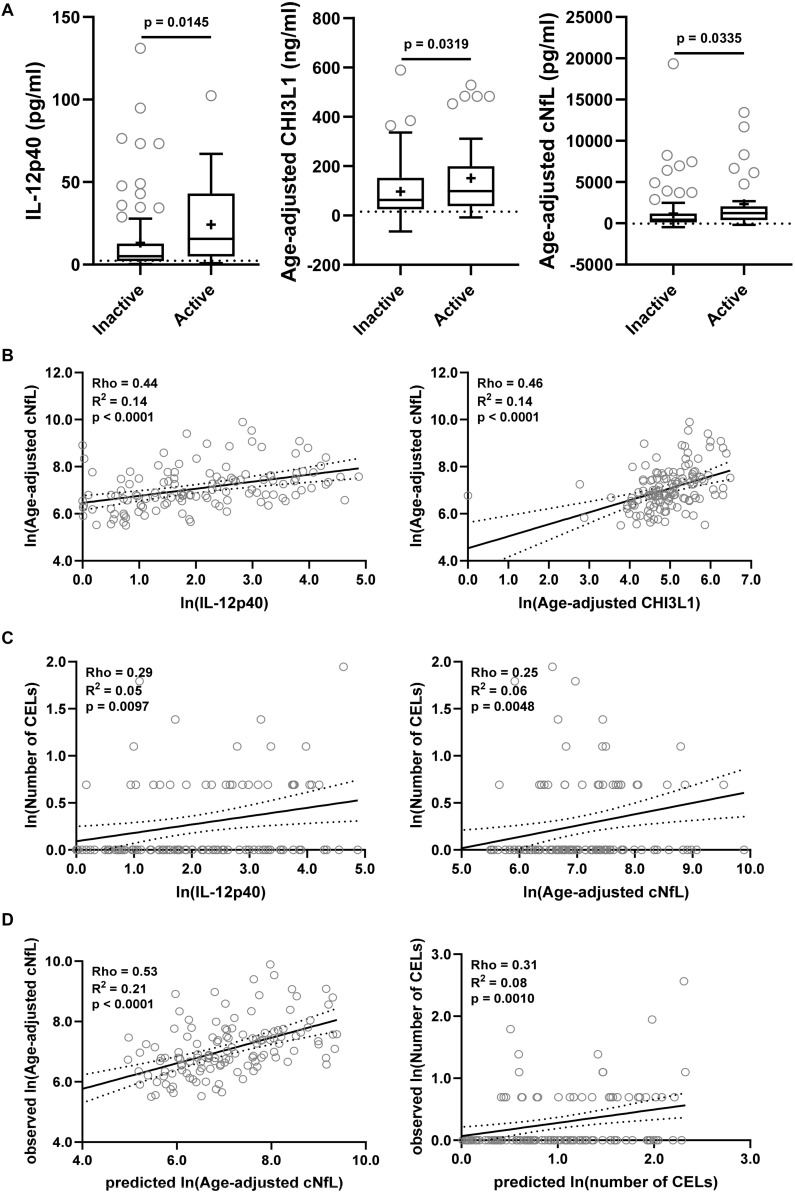
The findings from the training cohort with a stronger statistical effect (*p* < 0.01; IL-12p40, CHI3L1, and cNfL) were then validated in an independent validation cohort. **(A)** Biomarker concentrations were compared across lesional activity subgroups (inactive versus active, *n* = 96 and 34, respectively). Dotted line represents median of HDs and “+” sign represents mean of respective subgroup. **(B)** In MS patients (*n* = 130) correlations between cNfL and IL-12p40, CHI3L1 were analyzed using Spearman analysis. Linear regression of IL-12p40 and CHI3L1 with cNfL as a dependent variable was analyzed. Solid line represents “line of best fit,” and dotted line represents 95% confidence intervals. **(C)** In MS patients (*n* = 130) correlations between number of CELs and all biomarkers were analyzed using Spearman analysis; Only for statistically significantly (IL-12p40 and cNfL; *p* < 0.01) correlated biomarkers linear regression with number of CELs as a dependent variable was analyzed. **(D)** In validation cohort, the cNfL concentrations were predicted using combined linear regression model of IL-12p40 and CHI3L1 (from training cohort), and number of CELs were predicted using combined linear regression model of IL-12p40, CHI3L1 and cNfL. And then linear regression between predicted and observed values were analyzed.

The findings from the training cohort with a stronger statistical effect (*p* < 0.001; IL-12p40, CHI3L1, and cNfL) were then validated in an independent validation cohort (Figure 6). All three biomarkers were significantly elevated in MS patients with active lesional activity compared to inactive patients, but with a less strong statistical effect (IL-12p40, CHI3L1, and cNfL unadjusted *p* = 0.0145, 0.0319, and 0.0335, respectively; Figure 6A).

IL-12p40 (Rho = 0.44, *R*^2^ = 0.14, and *p* < 0.0001) and CHI3L1 (Rho = 0.46, *R*^2^ = 0.14, and *p* < 0.0001) were moderately but significantly correlated with cNfL (Figure 6B). While only IL-12p40 (Rho = 0.29, *R*^2^ = 0.05, and *p* = 0.0097) and cNfL (Rho = 0.25, *R*^2^ = 0.06, and *p* = 0.0048) were weakly but significantly correlated with number of CELs (Figure 6C).

Within validation cohort, the cNfL concentrations predicted using combined linear regression model of IL-12p40 and CHI3L1 (from training cohort) outperforms (Rho = 0.53, *R*^2^ = 0.21, and *p* < 0.0001) any biomarker discretely. Similarly, the number of CELs predicted using combined linear regression model of IL-12p40, CHI3L1 and cNfL is marginally better (Rho = 0.31, *R*^2^ = 0.08, and *p* = 0.0010) than any biomarker alone (Figure 6D).

## Discussion

We observed moderate but reproducible positive associations of IL-12p40 and CHI3L1 with MRI CELs and with cNfL, the marker of axonal damage. IL-12p40 (encoded by IL12B gene) is mostly produced by activated cells of myeloid lineage, such as monocytes, macrophages, microglia, neutrophils and myeloid dendritic cells, and induces Th1 polarization of T cells (Kanangat et al., 1996; Kichian et al., 1996; Cooper and Khader, 2007), while CHI3L1 is mostly produced by neutrophils and activated astrocytes in the CNS and plays a role in Th2 T cell polarization (Boesen et al., 2018). CELs exhibited a weaker positive correlation with pro-inflammatory mediators, TNFα (mostly produced by activated macrophages but also by T cells) and TNFβ (also called Lymphotoxin-α and secreted mostly by activated lymphocytes), and also with immunoregulatory cytokine, IL10 (secreted mostly by monocytes). Collectively these results point to a strong CNS activation of innate immunity at the time when CELs are visible. We can’t make any determination of causality from our study, although the indirect evidence for pathogenicity of CHI3L1 does exist in the literature: subjects with clinically isolated syndrome (CIS) who had high CSF levels of CHI3L1 had greater and faster transition to clinically definite MS and a four-fold increased risk for the development of neurological disability compared to CIS subjects with low CSF CHI3L1 levels (Comabella et al., 2010; Canto et al., 2015). Additionally, CHI3L1, in concentrations analogous to those measured in the active MS group in this study was mildly neurotoxic to primary cultured neurons *in vitro* (Matute-Blanch et al., 2020). The possibility of direct or indirect neurotoxicity of CSF biomarkers associated with CELs is supported by the positive correlation of CHI3L1 (as well as IL-12p40 and TNFα) with cNfL. In contrast, CSF concentrations of immunoregulatory cytokine TGFβ, secreted in the CNS mostly by resting microglia, was significantly, although mildly decreased in MS patients with CELs. This is likely due to activation of microglia and their phenotypical switch from an immunoregulatory toward pro-inflammatory phenotype. The strong validation of cNfL prediction using combined model of IL-12p40 and CHI3L1 suggests that MS lesional activity is major contributor to MS-associated axonal damage. This conclusion is supported by validity of combined model of IL-12p40, CHI3L1 and cNfL to predict MS lesional activity measured by number of CELs.

In the introduction, we alluded to the limitations of quantifying MS lesional inflammatory activity via MRI CELs: CEL numbers do not capture CEL volume and the visibility of CELs is dependent on dose of the contrast administered and the delay between contrast administration and image acquisition. From that standpoint, it is intriguing to observe that MS patients who lacked CELs still had elevated CSF levels of CEL-associated biomarkers: IL-12p40 (1.3-fold), CHI3L1 (5.6-fold), TNFα (1.6-fold), and IL10 (2.0-fold) in comparison to HD medians. This suggests presence of MS lesional activity that is not visible by CELs, either because it’s located in the CNS tissue not imaged (e.g., spinal cord), reflects formation of demyelinating cortical MS lesions which do not enhance, or is below the CEL detection threshold using a standard contrast dose.

Surprisingly, we found that other inflammatory CSF markers, especially those associated with adaptive immunity are elevated in both MS subgroups (i.e., with CELs or without) comparatively: CD27 (mostly secreted by T cells, CD8 > CD4), CD21 (secreted by naïve B cells), BCMA (secreted by plasma cells), and CXCL13 (chemokine secreted by follicular helper T cells and follicular dendritic cells that is B cell attractant). This observation was further supported by an analogous decrease in CSF BAFF, a fundamental survival factor consumed by B cells, found in both active and inactive MS groups. This was also true for a marker of toxic astrocytes, SERPINA3.

While the simplest interpretation of these observations is that cells of adaptive immunity do not play an essential role in the formation of MS lesions, this interpretation is clearly incorrect. Although pathological studies of active MS lesions have shown that in some instances the loss of oligodendrocytes and demyelination can occur in absence of adaptive immune cells, B and T lymphocytes (Barnett and Prineas, 2004; Henderson et al., 2009; Hernández-Pedro et al., 2013), and other studies have shown a predominance of activated myeloid and glial cells (monocytes, macrophages, microglia, and astrocytes) in acute MS lesions (Lucchinetti et al., 2000; Prineas et al., 2001; Howell et al., 2010; Mishra and Wee Yong, 2016; Moliné-Velázquez et al., 2016) the high therapeutic effect of B cell-depleting or lymphocyte-depleting treatments provides proof for the essential role of lymphocytes, especially B, cells in the formation of MS CELs.

So how can we explain this apparent discrepancy? We believe that the answer lies in the different topological distribution of cells of adaptive immunity in the early versus later stages of the disease: at the beginning of the MS process, when no lymphoid tissue has been formed in CNS, all cells of adaptive immunity concentrate in the active MS lesions. Once tertiary lymphoid follicles are formed in the meninges, then many cells of adaptive immunity reside in these lymphoid organs or are distributed across CNS tissue behind closed BBB. An alternative explanation is that cells of adaptive immunity become activated first and secrete factors that open BBB and recruit cells of myeloid lineage; by the time CELs are visible, the lymphocytes pass their expansion/activation status. This hypothesis is contradicted by observations stemming from a clinical trial of altered peptide ligand (APL) of myelin-basic protein (MBP) in MS, which demonstrated clear expansion of T cells in the CSF at the time of APL-induced CELs (Bielekova et al., 2000).

Finally, the last group of CSF biomarkers, comprised of IL1β, IL17F, GM-CSF, CD14, TRAIL, and LIF were not significantly elevated in either MS group. We note that first three of these are linked to Th17 T cells, which play a pathogenic role in animal model Experimental Autoimmune Encephalomyelitis (EAE). However, our data do not necessarily rule out the pathogenic role these cytokines may play in MS, because CSF levels of some were close to the detection limit of the applied assays (i.e., IL17F, LIF, and IL1β), while those with CSF levels clearly above the detection limits (i.e., GM-CSF and TRAIL) could have been consumed by activated immune cells.

Finally, we want to address differences between cNfL and sNfL as less invasive blood collection is preferred over spinal tap and advent of highly sensitive assay (Simoa^TM^) (Rissin et al., 2010; Kan et al., 2012) allows reproducible measurement of sNfL (Kuhle et al., 2016, 2017; Disanto et al., 2017; Siller et al., 2019). But multiple studies, including this one demonstrated some loss of accuracy and clinical utility of sNfL as compared to cNfL (Kuhle et al., 2016; Disanto et al., 2017).

Though sNfL levels were correlated with cNfL (Rho = 0.60, R^2^ = 0.34, and *p* < 0.0001) sNfL measurement did not differentiate between non-active and active MS lesional inflammatory activity subgroups with pre-defined statistical significance (cNfL: *p* = 0.0043 and sNfL: *p* = 0.0690). Similarly, sNfL levels were only moderately or weakly correlated with number of CELs (Rho = 0.30 and *p* = 0.011) and pro-inflammatory biomarkers associated with lesional inflammatory activity: IL-12p40 (Rho = 0.18 and *p* = 0.129) and CHI3L (Rho = 0.29 and *p* = 0.021). Whereas, cNfL levels were strongly correlated with both number of CELs (Rho = 0.62 and *p* < 0.0001) and biomarkers of lesional inflammatory activity: IL-12p40 (Rho = 0.55 and *p* < 0.0001) and CHI3L (Rho = 0.59 and *p* < 0.0001).

Correlation analysis between cNfL and sNfL explains just 34% of variance (*R*^2^ = 0.34); that leaves 66% of variance unexplained. To make sNfL more accurate, we need to identify possible confounding factors that will need to be mathematically adjusted for to strengthen correlation between cNfL and (adjusted) sNfL and thus enhance the clinical utility of the latter.

## Data Availability Statement

The original contributions presented in the study are included in the article/Supplementary Material, further inquiries can be directed to the corresponding author/s.

## Ethics Statement

The studies involving human participants were reviewed and approved by the National Institutes of Health (NIH) and Institutional Review Board (IRB). The patients/participants provided their written informed consent to participate in this study.

## Author Contributions

BB and RM designed the study. RM, JP, and MK performed the experiments. BB, RM, and TW analyzed the data. RM wrote the first draft of the manuscript. BB supervised all aspects of the study. All authors contributed to manuscript revision, read, and approved the submitted version.

## Conflict of Interest

MK contributed to this work as a former employee of NINDS, NIH, and the opinions expressed in this manuscript do not represent her current affiliation – Eli Lilly Japan K.K., Kobe, Japan. The remaining authors declare that the research was conducted in the absence of any commercial or financial relationships that could be construed as a potential conflict of interest.

## Supplementary Material

Supplementary Figure 1 In the training cohort biomarker concentrations were compared across MS disease type (RR-MS vs P-MS) within respective lesional activity subgroups (inactive and active) using unpaired *t*-test (Inactive: RR-MS and P-MS, *n* = 7 and 28, respectively; Active: RR-MS and P-MS, *n* = 28 and 7, respectively). “+” sign represents mean of respective group.

Supplementary Table 1 In the training cohort, within MS patients (*n* = 70) correlations between cNfL and other biomarkers were analyzed using Spearman analysis; Only statistically significant correlations (*p* < 0.01) are highlighted.

Supplementary Table 2 In the training cohort, within MS patients (*n* = 70) correlations between number of CELs and all analyzed biomarkers were analyzed using Spearman analysis; Only statistically significant correlations (*p* < 0.01) are highlighted.

Supplementary Data File 1 Sheet 1: biomarker concentrations (cNfL, sNfL, and CHI3L1) with their respective sample collection date, age, and gender for healthy cohort. Sheet 2 and 3: all analyzed biomarkers’ concentrations for all subjects with their respective sample collection and MRI dates, age, disease duration, gender, EDSS, MS disease type, lesional activity (Inactive or Active), and number of CELs, for both (training: sheet 2 and validation: sheet 3) cohorts.
