# Supplementary figures and images for: Cerebrospinal Fluid Biomarkers of Myeloid and Glial Cell Activation Are Correlated With Multiple Sclerosis Lesional Inflammatory Activity
Source: Front Neurosci. 2021 Mar 30;15:649876. doi: 10.3389/fnins.2021.649876 (PMC8042223; doi:10.3389/fnins.2021.649876)

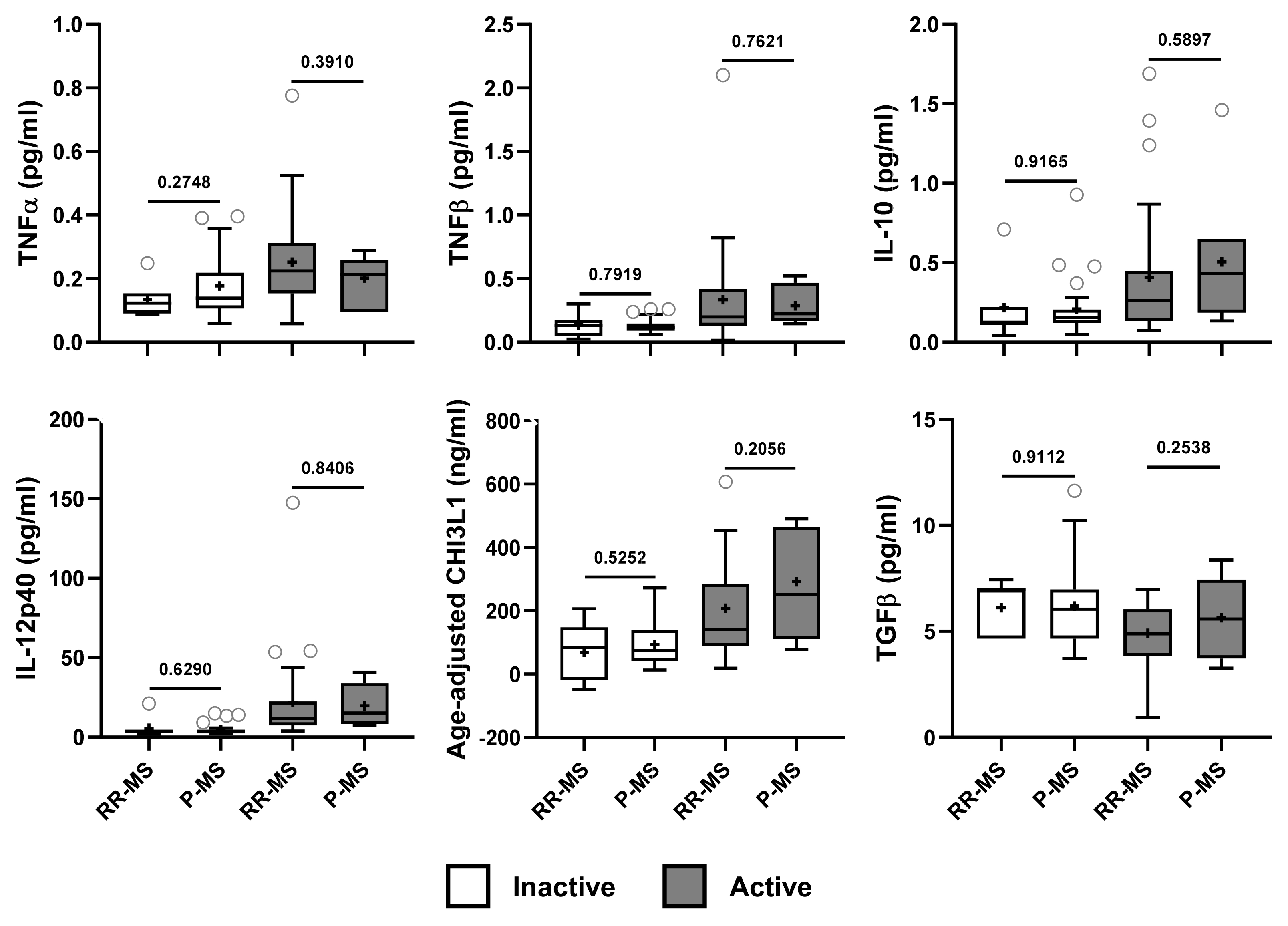

Supplement: Supplementary Figure 1 — In the training cohort biomarker concentrations were compared across MS disease type (RR-MS vs P-MS) within respective lesional activity subgroups (inactive and active) using unpaired t-test (Inactive: RR-MS and P-MS, n = 7 and 28, respectively; Active: RR-MS and P-MS, n = 28 and 7, respectively). “+” sign represents mean of respective group. [file Image_1.jpeg]
